# Martini 3 Coarse-Grained Model for Second-Generation Unidirectional Molecular Motors and Switches

**DOI:** 10.1021/acs.jctc.2c00796

**Published:** 2023-01-10

**Authors:** Petteri Vainikka, Siewert J. Marrink

**Affiliations:** † Zernike Institute for Advanced Materials, University of Groningen, Nijenborgh 4, 9747 AGGroningen, The Netherlands; ‡ Groningen Biomolecular Sciences and Biotechnology Institute, University of Groningen, Nijenborgh 7, 9747 AGGroningen, The Netherlands

## Abstract

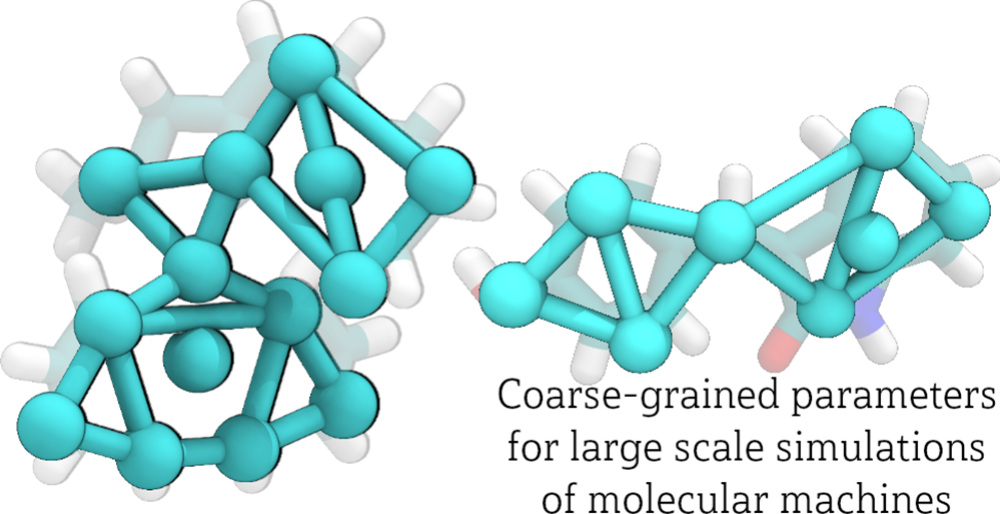

Artificial molecular motors (MMs) and switches (MSs), capable of undergoing unidirectional rotation or switching under the appropriate stimuli, are being utilized in multiple complex and chemically diverse environments. Although thorough theoretical work utilizing QM and QM/MM methods have mapped out many of the critical properties of MSs and MMs, as the experimental setups become more complex and ambitious, there is an ever increasing need to study the behavior and dynamics of these molecules as they interact with their environment. To this end, we have parametrized two coarse-grained (CG) models of commonly used MMs and a model for an oxindole-based MS, which can be used to study the ground state behavior of MMs and MSs in large simulations for significantly longer periods of time. We also propose methods to perturb these systems which can allow users to approximate how such systems would respond to MMs rotating or the MSs switching.

## Introduction

The past two decades have seen an increasing amount of studies dedicated to synthesis and applications of light-driven molecular motors (MMs), capable of unidirectional rotation, typically when activated with UV light. After the first successful synthesis of these motors in 1999 by Ben Feringa and colleagues,^1^ MMs have been applied to perturb cellular membranes in order to induce necrosis and to transport chemical components to the cell,^2^ perform on-demand release of calcein from liposomes,^3^ induce differentiation of stem cells,^4^ and even create muscle-like supramolecular assemblies capable of photoactuation.^5,6^

Some of the applications of MMs demand that they be functionalized. Examples can be found in the work of Tour et al.^2^ where the motors were fitted with specific peptide sequences to target certain cells, or in the work of van Rijn et al.^4^ where the motors had been fitted with two isophtalic acid groups in order to induce electrostatic anchoring on a charged surface.

There are no strict rules for classifying MMs, but they can be divided into various generations depending on the amount of stereogenic centers; the first-generation molecular motors contained two stereocenters, the second-generation motors contain only one, and the most recent third-generation motors contain none.^7^

Likewise, light-driven molecular switches (MSs) have garnered a significant amount of attention in both materials science and bioscience. Multiple classes of MSs exist, with azobenzene derivatives being the most actively studied class.^8^ These switches, as their name suggests, operate by using UV or visible light to reversibly switch between isomeric states. The effect of this switching need not be limited to the conformational change, as some switches change their physicochemical attributes as they undergo irradiation.^9^

Besides the various experimental applications, there are numerous spectroscopic and theory-based studies of MMs and MSs. These studies have, among other things, predicted potential new motors^10,11^ and switches,^12^ shed light on the mechanics of rotation of existing MMs,^13−17^ and further characterized photophysical properties of MSs.^18,19^

Due to the complex and intricate way the rotors and switches function when excited, there are only a limited number of studies that utilize classical molecular dynamics (MD).^20−25^ This is unsurprising, but also hints toward a prevailing mindset; most applications seem to work under the premise that MMs/MSs primarily affect the studied systems by undergoing unidirectional rotation or switching—something which classical MD methods can hardly capture. While this assumption is probably true in many cases, in order to establish a rigorous causal link between effects seen in experiments and the rotation/switching, there needs to be a way to study other possible effects MMs and MSs might have on their surroundings. As the experiments conducted with MMs and MSs are becoming more complex and involving larger quantities of biomolecules, such as proteins and membranes, the use of classical all-atom (AA) simulations is becoming less feasible due to the high computational costs.

As an alternative, coarse-grained (CG) simulations are widely used, allowing larger spatiotemporal scales to be explored by omitting some of the atomistic degrees of freedom.^26^ In particular, the CG Martini model has become popular for biomolecular simulations, including an increasing amount of studies on the interplay between synthetic and biological molecules.^27,28^ With the recent release of the Martini 3 model,^29^ featuring improved interactions between small molecules and their environment in general,^30^ we anticipate a need for well-designed and validated topologies for the basic units that make up MMs and switches, which would enable large-scale studies of how these molecules might affect their surroundings.

**Figure 1:**
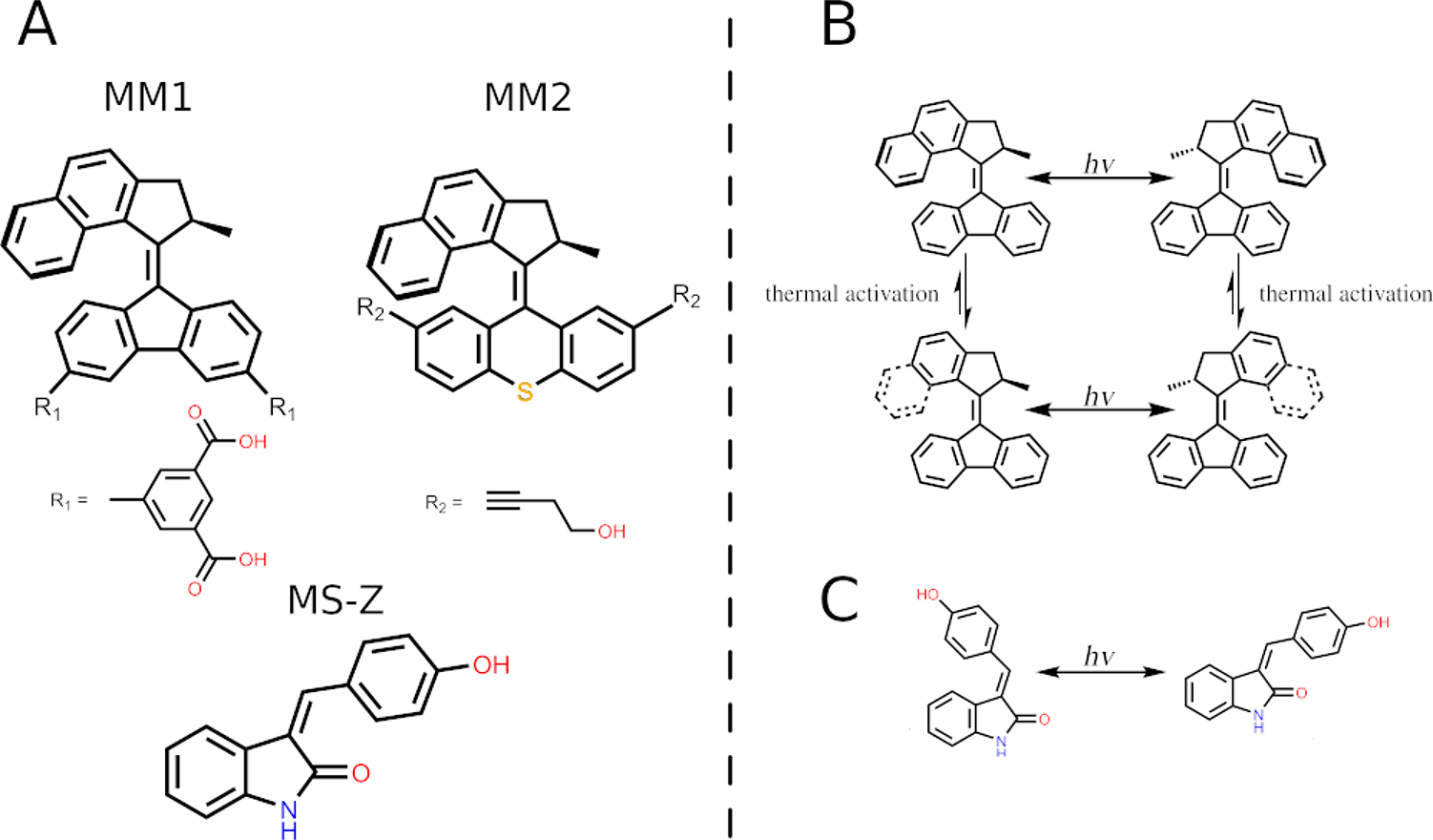
(A) Structures of molecular motors (**MM1** and **MM2**), their addends (**R**_**1**_ and **R**_**2**_), and a molecular switch (**MS-Z**) parametrized in this work. Only the *Z*-stereoisomer is shown for the molecular switch. Addends, when present, are always added so that the symmetry of the stator is preserved. (B) Activation scheme of the MM. (C) Activation of the MS.

Therefore, in this work we present two Martini 3 models^29^ for second-generation MMs and two functionalized versions of those MMs, (FMMs). Additionally we provide a CG model for an oxindole-based MS which is currently being applied in experimental studies alongside with MMs. These molecules are illustrated in Figure 1. It should be noted that due to the structural similarity of the various generations of MMs, our models can easily be extended to the other two generations. The models are validated with respect to AA reference simulations, as well as to cheminformatic predictions on their partitioning free energy in octanol.

The remainder of this work is structured as follows: The first section considers parametrizing CG models from the higher resolution AA models. Following the parametrization we go through several validation procedures to ensure the derived CG models are capable of reproducing computationally predicted behavior such as stacking and partitioning. Finally, we show a few potential applications of MMs in biological environments and propose some approaches on how to incorporate rotation and switching behavior to CG simulations.

## Methods

### Computational Details

All simulations were performed with GROMACS 2021.3.^31,32^ Temperature of the simulation was controlled using the Bussi–Donadio–Parrinello thermostat (V-rescale).^33^ Equilibration simulations used the Berendsen barostat,^34^ and all production simulations were performed using the Parrinello–Rahman barostat.^35^ Biphasic systems and bilayer containing systems used a semi-isotropic pressure coupling, and all other systems used an isotropic pressure coupling. In both cases, the reference pressure was set to 1.0 bar and the compressibility was set to 3.0 × 10^–4^ bar^–1^. Constraints were solved using LINCS,^36^ with a LINCS order of 4. All-atom production simulations were run with a 2 fs time step, while the CG production simulations used a 20 fs time step. Electrostatic interactions in AA systems were computed using the smooth particle mesh Ewald (PME) method,^37^ whereas CG systems used the reaction-field method. Enforced rotation, required to mimic the rotation of MMs, was performed with the “pivot free” isotropic potential (rot-type0 = iso-pf), with no mass weighting, rotational rate of 80°/ps and a force constant of 280 kJ/(mol nm^2^). The switching behavior of MSs was mimicked simply by continuing a previous simulation with a new molecular topology assigned for the MS, corresponding to the other stereoisomer.

### Simulation Setups

AA reference simulations of the MMs and MS were set up in identical ways. First, initial GROMOS54a7 topologies were obtained from the ATB web server^38−40^ and further optimized using Q-Force^41^ in conjunction with Gaussian 16.^42^ All topologies used in this study can be found in the Supporting Information. Subsequently, the atomistic model was placed in an empty 3.5 × 3.5 × 3.5 nm^3^ simulation volume, in which it was solvated using the SPC water model. The system energy was minimized using the steepest-descent algorithm and then equilibrated in three steps: A 250 ps simulation in a *NVT* ensemble with temperature set to 103 K, followed by another 250 ps in the same ensemble and ambient temperature (300 K), and finally a 1 ns simulation in ambient temperature within the *NpT* ensemble. Equilibration was followed by a production run of 1 μs, again in the *NpT* ensemble and at ambient temperature. Atomistic references for both stereoisomers of the MS were simulated separately. CG simulations of individual components started by placing the target compound in a 5 × 5 × 5 nm^3^ simulation volume, which was then solvated with regular Martini water. Minimization was carried out in the same way as that with the AA systems. Equilibration for each system was done in five steps, during which the time step was increased gradually from 1 to 20 fs, and the temperature is brought up to 300 K from 200 K.

Biphasic water–octanol systems, required for estimating the log *P* by using umbrella sampling (US), were built by equilibrating a cubic box of octanol and then elongating it such that *L*_*x*_ = *L*_*y*_ < *L*_*z*_. The remaining empty volume was then filled with water. The system was equilibrated for 1 μs to fully saturate the octanol phase with water. The target component was then inserted in the aqueous phase, after which the whole system was re-equilibrated.

Hydrated octanol systems, required for estimating the Δ*G*_oco_ → vac during the thermodynamic integration (TI) routine, were built by randomly placing water and octanol molecules in a cubic volume such that the mole fraction of water was 0.2.

Additionally, we studied dimerization propensities of both MMs using US. Two MMs were placed in a 7 × 7 × 7 nm^3^ simulation volume which was then solvated with either regular Martini water or SPC water, depending on the resolution. After solvation, AA systems were equilibrated for 10 ns and CG systems for 500 ns.

For showcasing the potential applications of our models, we recreated two experimental system setups: One with MMs electrostatically anchored on to a inorganic surface^4^ and one in which MMs are embedded to a lipid bilayer.^2^ The inorganic surface test case presented a slight challenge, as there are currently no models available for Martini 3 which could be used to describe such a surface. In lieu of undertaking the rather significant task of parametrizing an accurate surface model, we chose to create a very simplified surface model which would subsequently be coated with positively charged TQ4p beads, which can be used to sufficiently represent the amine coating used by Zhou et al.^4^ We formed a slab surface by creating a bead which has a strong self-interaction, a favorable interaction with TQ4p, and a slightly repulsive interaction with everything else. A surface made from these beads was subsequently fully coated with TQ4p beads, carrying a positive unit charge. The surface was deemed to be fully coated once the addition of more TQ4p beads resulted in the beads remaining in the solvent phase, instead of adsorbing to the surface. After the preparation of the coated surface, we introduced FMM1 to the solvent phase above the surface. FMM1 has two isophtalic acid groups which both carry a net charge of −2, allowing it to electrostatically anchor on the positively charged surface. We compared the behavior of FMM1 on the simplified surface model to an AA model. Unlike the CG surface model, the AA model has the R–NH_3_^+^ groups covalently bound to each Si atom on the surface, creating a full and uniform coating. The AA surface is also completely flat; the CG model can be tuned to recreate such a topology if needed, but it is the understanding of the authors that surfaces such as those used by Zhou et al.^4^ are not atomistically flat, so we allowed the surface to deform slightly. The full details of the AA system have been previously published elsewhere.^4^

The biological assembly setup was remarkably more straightforward: A DOPC bilayer was created on the *xy* plane of a *z*-elongated simulation box using the *insane* tool.^43^ We parametrized a model for calcein in physiological pH (7.5), placed it inside the simulation volume, and pulled it through the bilayer using a US protocol described later in this document. To quantify the effect which FMM2 might have on the translocation of calcein, we created systems with 16, 32, and 50 FMM2 embedded in the bilayer and repeating the US step. The embedding of FMM2 in the bilayer was performed with free sampling, by placing FMM2 to the solvent phase and allowing it to diffuse to the bilayer.

### Parameterization Strategy

Building of the CG models began by deriving bonded interactions from the forward mapped AA simulations. The resulting preliminary models were then simulated for 1 μs in water at the CG level, after which their bond, angle, and dihedral profiles were compared to the mapped AA counterparts. This process was iterated until a sufficiently good match was obtained. Nonbonded interactions, dictated by the selection of bead types, were derived from either previously established Martini 3 models^30^ or from the detailed Supporting Information of the Martini 3 work^29^ (pp 19–47). The correctnesses of these parameters were tested by estimating the water–octanol partition coefficient (log *P*) for each compound. These values were compared against other predicted values and experimental log *P* values of structural analogues (where available).

As is common for Martini 3 models, we use the solvent accessible surface area (SASA) to semiquantitatively estimate if the chosen mapping of the compound produces a reasonable shape and size at the CG level. This is performed with the built-in *gmx sasa* tool, found in the GROMACS software. The bead radii for Martini 3 beads are known, and we use values derived by Rowland and Taylor^44^ to approximate the sizes of the atoms in the AA model.

#### Free energy estimates, partition coefficients and dimer formation

Estimation of the water–octanol partitioning coefficient was performed with umbrella sampling (US) and thermodynamic integration (TI).

The TI method for estimating the free energy of solvation in water and water–octanol mixture (20% water, 80% octanol) was conducted by running three sets of simulations, each with 21 steps during which the nonbonded interactions between the solute and solvent were scaled from full to none. A Δ*G* value was compiled from the results, using Bennet’s acceptance ratio (gmx bar). Soft-core potentials were applied with sc-α = 0.5 and sc-p = 1. Temperature was set to 310 K. Finally, the partition coefficient was estimated by
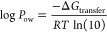
1

US simulations were performed with the same settings and time steps as previously described. COM of the target solute (MM or MS) was pulled away from COM of water along the *z*-axis with 0.1 nm steps, with a force constant of 1500 kJ mol^–1^ nm^–2^. Each window was for 1 μs. Analysis was performed using *gmx wham*, with 300 bins and 100 bootstraps to estimate uncertainty. Dimer formation of MMs was studied on both AA and CG resolutions using the same approach, with the exception of using a smaller overall range of 0.3–2.0 nm, and performing the pulling along all the Cartesian coordinates. No orientational restraints were implemented, and thus the motors could freely adopt any orientation in relation to each other during the pulling.

## Results and Discussion

### Parameterization

**Figure 2:**
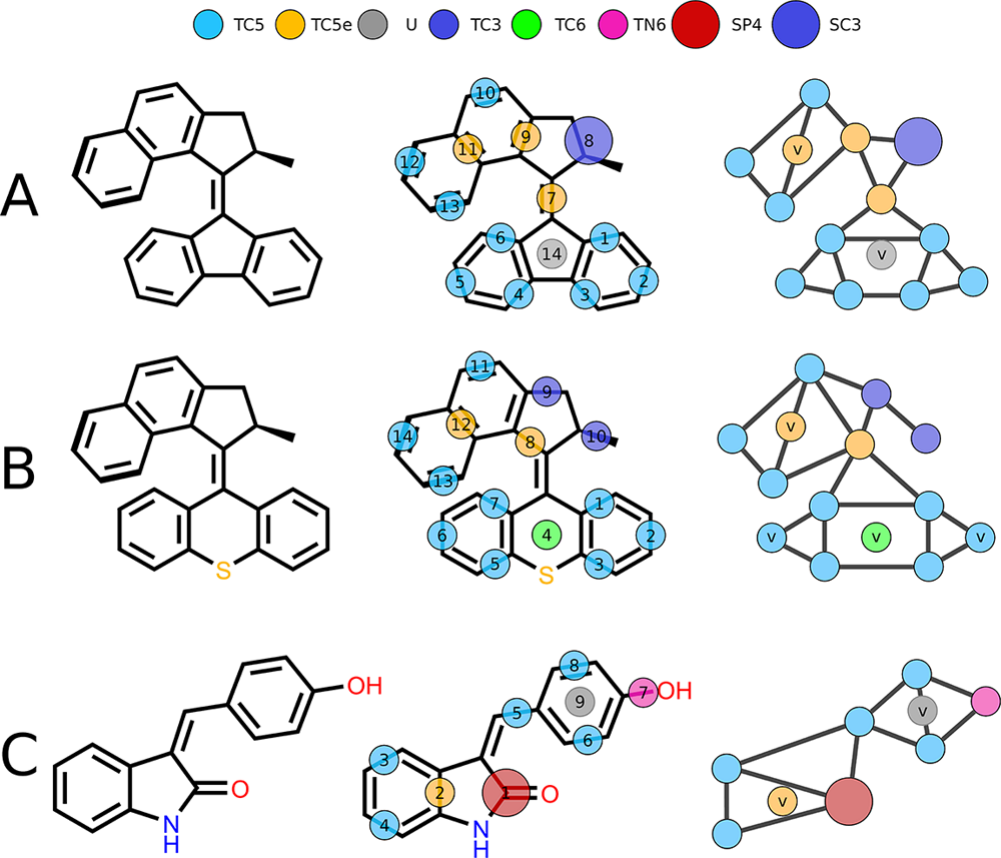
Mapping of CG motors and switches. Structural representation, mapping scheme, and final CG model of (A) **MM1**, (B) **MM2**, and (C) **MS-Z**. The bead assignment of **MS-E** (not shown here) is identical to the *Z*-stereoisomer. Virtual sites are indicated with the letter “v”. Parameters of all components are presented in the Supporting Information, Section 1.

The final mapping and bead type assignment of the MMs and MSs considered in this study are shown in Figure 2. The compounds presented in this work contain a lot of conjugated fused ring systems which can be accurately represented with the use of tiny (T) beads, as is showcased in the recent publication regarding Martini and small molecules.^30^ Small (S) beads were used only in two cases: to represent the acetamide moiety of the MS, in which the comparatively bulky and polar moiety can be well-represented by a SP4 bead; the other case being the methylcyclopentane group in the rotor of MM1. Due to slightly different stators between MM1 and MM2, we were not able to create a representative CG model of MM2 with an identical mapping and had to map the methylcyclopentane as TC3 beads. Virtual sites (v-sites) were used in all models: in MM1 the central bead of the naphthalene moiety (no. 11) is represented by a virtual TC5e bead, to keep the mapping of the moiety in line with a previously published Martini 3 naphthalene model.^30^ The other v-site in MM1 lies in the central 5-ring of the stator (no. 14) and is used to define a dihedral angle between the stator and the rotor. MM2 has a total of 4 v-sites, three in the stator and one in the naphthalene fragment. Two of the v-sites in the stator are used to represent the peripheral carbons of the stator (nos. 2 and 6), greatly simplifying the assignment of dihedral terms in the stator. Finally, there is a virtual TC6 bead in the middle of the stator, representing the sulfur group. The MS has only one non-interacting v-site, which is used to define a dihedral angle between the phenol and the oxyindole fragment. FMMs were parametrized similarly to MMs; a corresponding AA model was first refined using Q-force,^41^ followed by the same parametrization steps as described earlier in this work. This strategy was chosen as the additional groups in FMM1 and FMM2 were relatively small—future work with larger addends will likely require a fragment-based approach in which a thorough QM-based scanning is only performed for the MM—addend coupling sites. Additionally, we parametrized a model for calcein, which was used in one of the applications presented later in this work. The parameters and mapping for calcein are presented in Supporting Information Section 1.7.

We optimized the bonded parameters of the CG model by iteratively matching bond, angle, and dihedral distributions from mapped AA and CG simulations. The overall performance of the final CG models was deemed to be acceptable; the mean absolute error of bond lengths and unimodal angles between resolutions was 0.0025 nm and 5.5°, respectively. Bimodal angles, simulated with a quartic potential, had a MAE of 11.4°. Key parameters that dictate the overall configuration freedom of the compounds fully, such as dihedral profiles, were matched well between the two resolutions. These profiles can be found in the Supporting Information, Section 3. A further validation was performed by comparing the solvent accessible surface areas (SASAs) of AA models against their CG counterparts. The average difference between the two resolutions was 3.5%, with the largest individual difference being less than 6%—well within the acceptable range. All SASA values are reported in the Supporting Information, Section 2.

**Figure 3:**
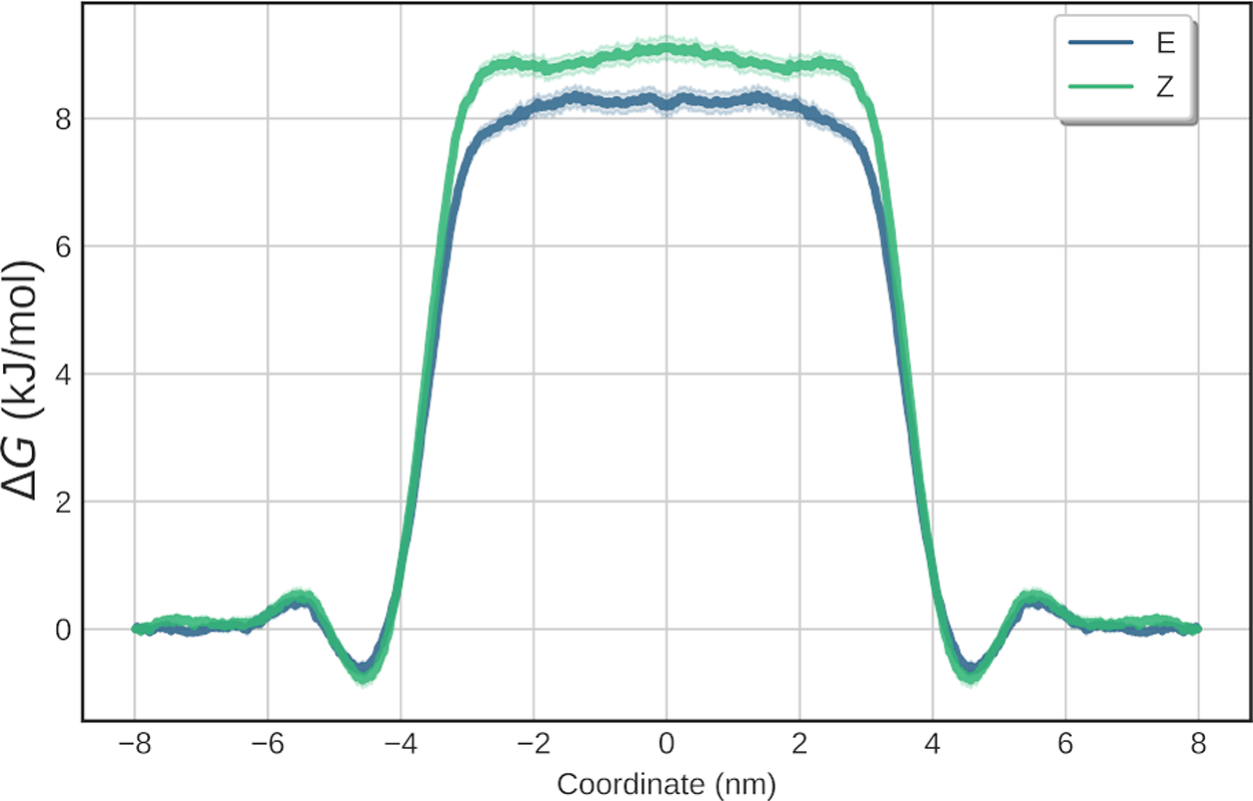
Water–octanol partitioning of both isomers of the MS. The aqueous phase lies between −4 and 4 nm. Although both stereoisomers are identical in terms of bead types, the *Z*-stereoisomer has a slightly higher preference to the octanol phase, as indicated by the higher potential on the aqueous phase.

Correctness of nonbonded interactions was evaluated by estimating the log *P* (water–octanol partition coefficient) of each CG model. Unfortunately there are no reported experimental log *P* values for any of the components presented in this work, and we had to resort to comparing our predicted values against values predicted by other software, in this case XLogP3^45^ and ALOGPS2.1.^46^ These values are given in Table 1. Comparison to atomistic data was omitted due to known issues with significant overestimation of the log *P* values.^47^ A second noteworthy detail was discovered while performing US on the two stereoisomers of the MS. Although comprised of the same beads, the polar acetamide moiety is shielded better in the *Z*-conformation, resulting in a slightly higher change in free energy of transfer between water and octanol, as can be seen in Figure 3. The log *P* of calcein (not listed in Table 1) was estimated to be −4.28, which is in good agreement with the reported experimental value of −4.04.^48^

**Table 1:** log *P* Values Obtained with Martini 3, ALOGPS2.1, and XLogP3^a^

	water–octanol partition coefficients		
compound	Martini 3	ALOGPS2.1	XLogP3
MM1	8.25 ± 0.05	7.22	7.49
FMM1	omitted	N/A	N/A
MM2	8.15 ± 0.06	7.84	8.04
FMM2	6.74 ± 0.15	6.96	6.19
MS-E	1.39 ± 0.05	3.10	2.54
MS-Z	1.53 ± 0.03	3.10	2.54

a Simulation results are at 310 K.

### π–π Stacking and Dimerization of MMs in Water

π–π stacking is known to be a driving force in the stacking of anthracene and anthracene-like compounds,^49^ as well as in fluorenes.^50^ As both structural motifs are present in our models, we set out to test how well our CG model captures the stacking effects in comparison to the AA models, which are known to be capable of consistently capturing dimerization free energies over multiple force fields.^51^ This effect can be probed in a straightforward fashion by comparing dimerization PMF profiles between the AA and CG models. We set up an US protocol with 18 windows, ranging from a distance of 0.3 to 2.0 nm between the COM of the motors. We then compared the overall energy of dimerization, as well as the most prominent dimer conformations predicted at both resolutions.

**Figure 4:**
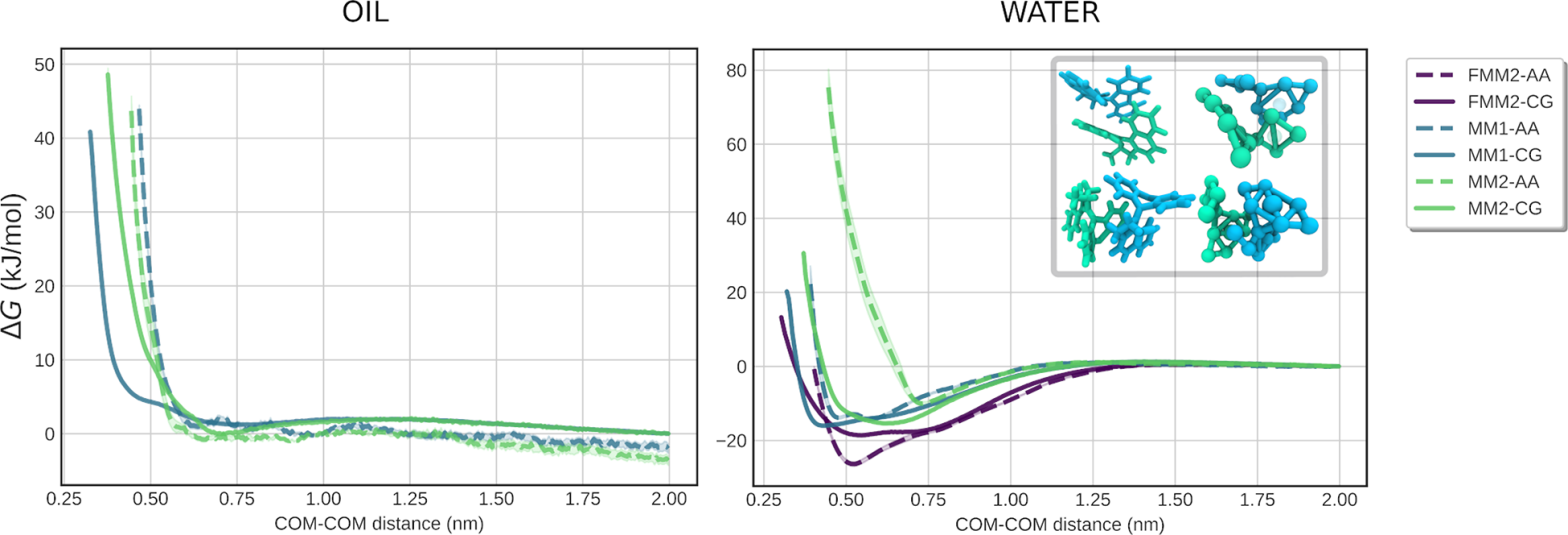
Left: PMF profiles describing the dimerization of **MM1**, **MM2** in octane. Right: PMF profiles describing the dimerization of **MM1**, **MM2**, and **FMM2** in water. PMFs were calculated by pulling the COMs of two MMs toward in steps of 1 Å. Inset: Most stable binding poses of **MM1** (top) and **MM2** (bottom).

Taken together, our models predict very similar stable (long-lived) dimer configurations as the atomistic resolution, and match the free energies at least qualitatively, as shown in Figure 4. This stands to reason, as the nonbonded interactions of T-beads in Martini 3 have been optimized to reproduce correct stacking distances of aromatic moieties.^29^

Studying dimerization of MM1 in water, we observe minute differences between the two resolutions; the minimum predicted by the CG model is located at 0.55 nm, whereas the AA model predicts it to be at 0.45 nm. The energy difference between the minima is 1.7 kJ/mol.

MM2 had more clear differences; the energy difference between the predicted minima is approximately 5.4 kJ/mol, and the CG model is able to pack more tightly, resulting in an approximate 0.3 nm shift in the profiles. The weakest performance is observed during dimerization for FMM2: The positions of the minima are within 0.1 nm of one another, but the CG model underestimates the lowest energy binding mode by approximately 8 kJ/mol. Since both MM1 and MM2 are very hydrophobic, their dimers are expected to be much more stable in water, with the hydrophobic effect further enhancing the stabilizing effect of π–π stacking. To get a more thorough understanding of the real π–π stacking propensity of MM1 and MM2, we performed simulations of dimerization in octane. We observe a distinct change in the profiles when compared to water: The overall shape before the repulsive regime is much more flat, with significantly shallower minima when compared to the same profile in water. This suggests that MM1 and MM2 have a significantly reduced dimer lifetime in octane and generally less propensity to dimerize. All in all, the CG models for the MMs and MSs considered in this work appear to be of good enough quality to allow applications involving more complex environments. In the next section, we explore a few potential directions.

### Potential Applications

#### Surface Anchored Molecular Motors

**Figure 5:**
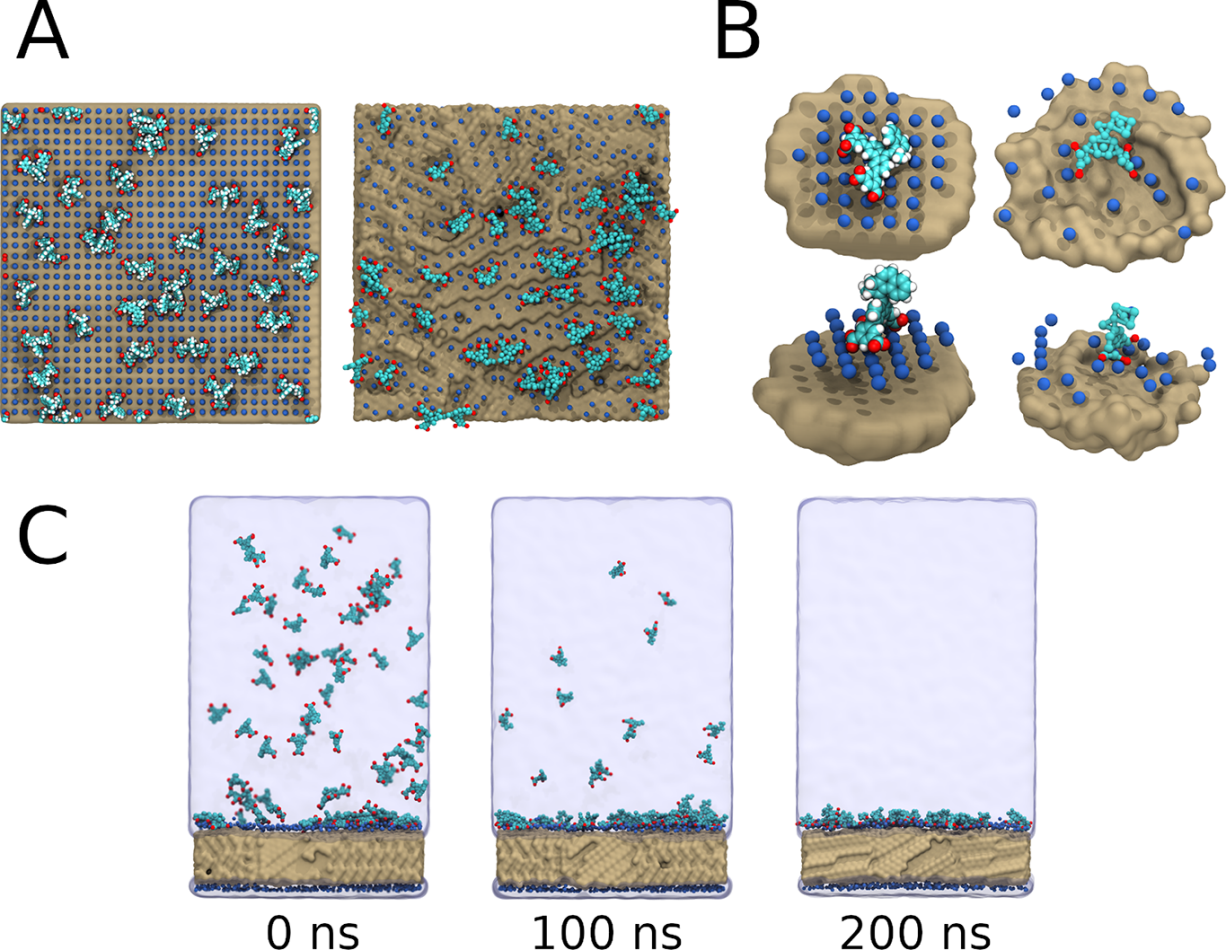
Surface anchored MMs. (A) Left: All-atom simulation with FMM1 anchored on a coated silica surface, water not drawn. Right: Similar system on the CG resolution. (B, Top) AA and CG models lying on the surface in a flat orientation. (Bottom) AA and CG models in a upright position on the surface. (C) Snapshots depicting the CG model of FMM1 anchoring on to the surface.

Previous and current work on MMs utilize inorganic surfaces in which the MMs are either adsorbed or anchored via electrostatic interactions. We set out to test whether our Martini 3 models could potentially be used for supplementing such studies in the future by parametrizing FMM1 and studying its interactions with an amine coated surface. Unfortunately there are no published Martini 3 models for silica surfaces, so we resorted to creating a simplified model, which consists of a layer of inert beads, coated with positively charged methylammonium groups which functionalize the surface (see Methods). Once fully coated, we added FMM1 to the solvent phase and allowed it to anchor on the surface. At the end of 200 ns of simulation, all MMs were attached to the surface, stabilized by the electrostatic interaction between the positively charged ammonium coating and the negatively charged isophtalic acid groups of FMM1 (Figure 5C).

To further validate our model, we compared the orientation of the anchored MMs to an AA simulation of the same system, as illustrated in Figure 5. We observed approximately 15% of the MMs lying flat on the surface (rotor pointing along the surface normal) at the AA resolution, whereas the CG system had approximately 30% of the MMs flat on the surface. There are a number of parameters that one can optimize, starting with the creation of a physically accurate model for a silica surface, but our toy model approach already indicates that CG simulations of systems such as those studied by Zhou et al.^4^ are possible. In short, it appears CG simulations can be used in the future to study the interactions of biomolecules (e.g., proteins) with surfaces and surface anchored MMs.

#### Interaction of MMs with Biological Assemblies

**Figure 6:**
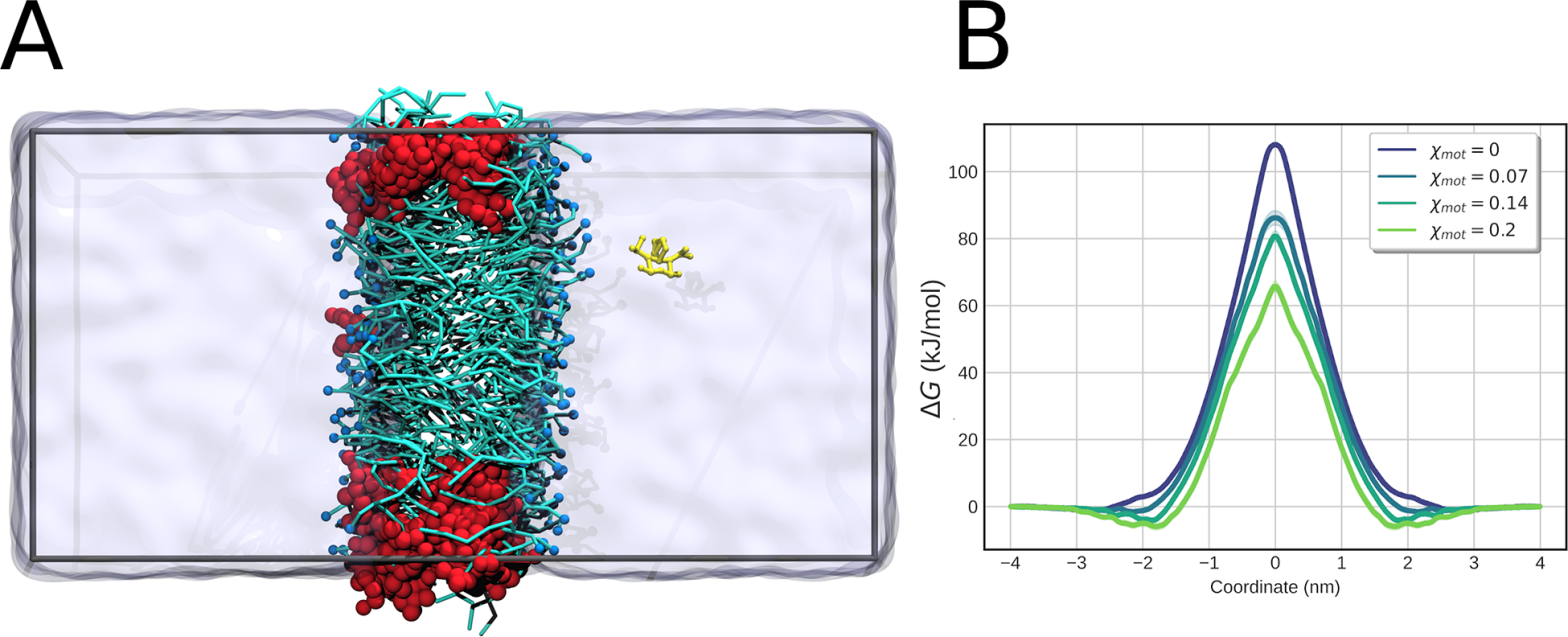
Effect of FMM2 on calcein permeability through DOPC bilayer. (A) Simulation setup. Calcein (yellow) is slowly pulled through the DOPC bilayer (cyan and blue), which contains FMM2 (red). The system is solvated in water (transparent surface). (B) Results of the tests. A significant depression in the potential of mean force is observed as χ_(FMM2)_ is increased gradually from 0 to 0.2.

Many of the experimental studies conducted with MMs take place in systems that include biomolecular entities such as lipid bilayers^2^ and proteins.^4^ We envision that our models will help supplement these studies by giving molecular level details of the behavior of MMs (and MSs) within these systems, and help explain the role which MMs have in them. As an example, FMM2 has been previously used in experimental work to perturb lipid bilayers and perform on-demand release of BODIPY dye from liposomes.^2^ Similar experimental work was subsequently performed with calcein.^3^ The working hypothesis presented in the literature appears to rely on the assumption that the UV-light–activated rotation of MMs perturb the bilayer and potentially opens nanoscale pores on the bilayer,^2,3^ resulting in the release of the dye. To test out our model, we set out to see how the presence of FMM2 in a simple DOPC bilayer would affect calcein permeation. In particular, we computed the PMF for translocation of calcein as a function of the mole fraction of FMM2. Without any perturbation, such as rotation of the MMs, we observe a significant depression in the barrier height as the mole fraction of FMM2 is increased (Figure 6), hinting that the MMs have a passive effect that should be quantified to better understand the on-demand release of dyes and other compounds from liposomes.

#### Approaches to Mimic Rotation

**Figure 7:**
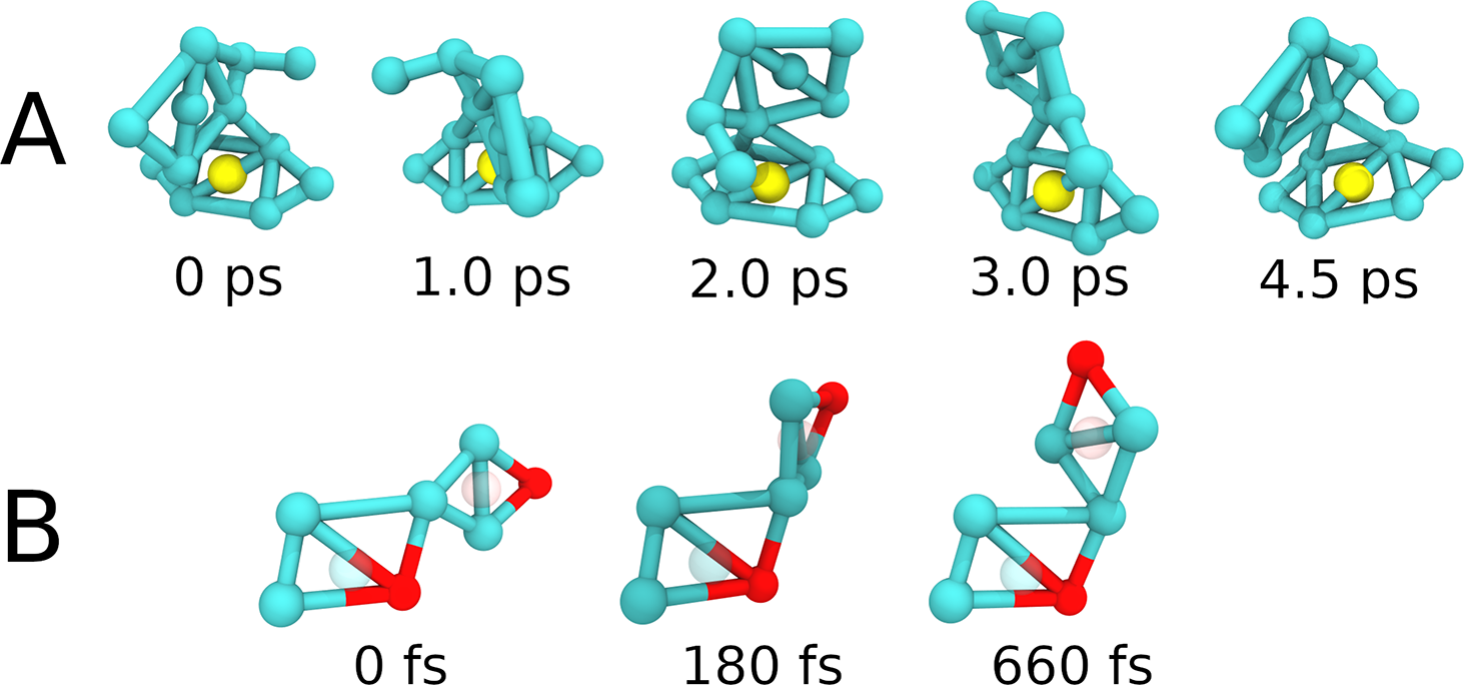
Toy model approach to rotation and switching behavior. (A) Enforced rotation of MM2, shown in 5 sequential snapshots that depict a full turn around the axle connecting the rotor and the stator. The enforced rotation was set up such that the MM will complete a full turn in approximately 4.5 ps. (B) MS switching from Z to E state, with one intermediate step. The switching process is almost instantaneous, only taking less than a picosecond.

Accurate modeling of the light activation of MMs and MSs remains a significant challenge for standard classical simulations. Previous work on MMs has indicated that the rotational rate of UV-activated MMs is quite limited in a laboratory setup and would require immense amounts of UV radiation to reach the ideal MHz range rotational rates.^52^ This would make these events extremely rare in our simulations which only span to μs time scales. In light of this, we settled with a toy model approach; using a standard non-equilibrium method implemented in GROMACS, *enforced rotation*, we can obtain very qualitative ideas on how a motor might affect its surroundings by undergoing light-activated rotation. The method allows the user to control the rate of rotation, as well as the force constant associated with it, giving the user freedom to tailor the rotation event to some extent. We envision this crude approach could potentially be used as a zeroth-order approximation to test how, for example, a lipid bilayer might react to a MM undergoing rotation while embedded in it. A significantly simpler strategy can be applied to the MSs by manipulating their molecular topology and forcing the MS to undergo a conformational change from *E* isomer to *Z*, or vice versa. Both the enforced rotation of MMs and the topological manipulation of MSs are depicted in Figure 7.

It should be noted that there are QM/MM methods^53^ which allow for much more accurate treatment of the rotation, although at the cost of significantly reducing the simulation size and time. However, with backmapping tools such as *backward*,^54^ we are able to convert our CG simulations back to AA level. This can potentially allow for a division of labor, in which the comparatively cheap CG simulations are used to self-assemble and/or equilibrate a MM containing system from which a relevant section can then be backmapped to be studied on a finer resolution.

## Conclusions

We have parametrized CG models of commonly used MMs and built a well-functioning model of a oxindole-based MS. The building-block approach used in the parametrization of these models allows for relatively easy manipulation of the topologies, and as more detailed thermodynamical data comes available, these models can be further refined accordingly. Combined with the large library of existing Martini 3 compounds, such as lipids, small molecules, proteins, etc., the new MM and MS models can be applied to a large variety of systems. Structures and parameters of these compounds will be uploaded and available in the Martini Database (https://mad.ibcp.fr).^55^

## Supporting Information Available

Model data and parameters, comparison of bonded terms, and SASA numerical data (PDF)
