# Supplementary material for: Martini 3 Coarse-Grained Model for Second-Generation Unidirectional Molecular Motors and Switches
Source: J Chem Theory Comput. 2023 Jan 10;19(2):596–604. doi: 10.1021/acs.jctc.2c00796 (PMC9878727; doi:10.1021/acs.jctc.2c00796)
Supplement: Supplementary file 1 — ct2c00796_si_001.pdf [file ct2c00796_si_001.pdf]

Martini 3 coarse-grained model for second-generation molecular motors and switches
Supplementary Information

Petteri Vainikka[1] and Siewert J. Marrink[1]*

[1]Groningen Biomolecular Sciences and Biotechnology Institute and Zernike Institute for Advanced Materials, University of Groningen, Nijenborgh 7, 9747 AG Groningen, The Netherlands

* Corresponding Author
E-mail: s.j.marrink@rug.nl (S.J.M.).

Number of pages: 18
Number of figures: 7
Number of tables: 19

1. Coarse-grained models parameterized for this study

Descriptions and illustrations of each compound created for this study is given below. We present a schematic representation of the compounds, accompanied by the mapping scheme, and bead assignment. The topology of each compound is given below the illustration.

1.1. Molecular motor 1 (MM1)

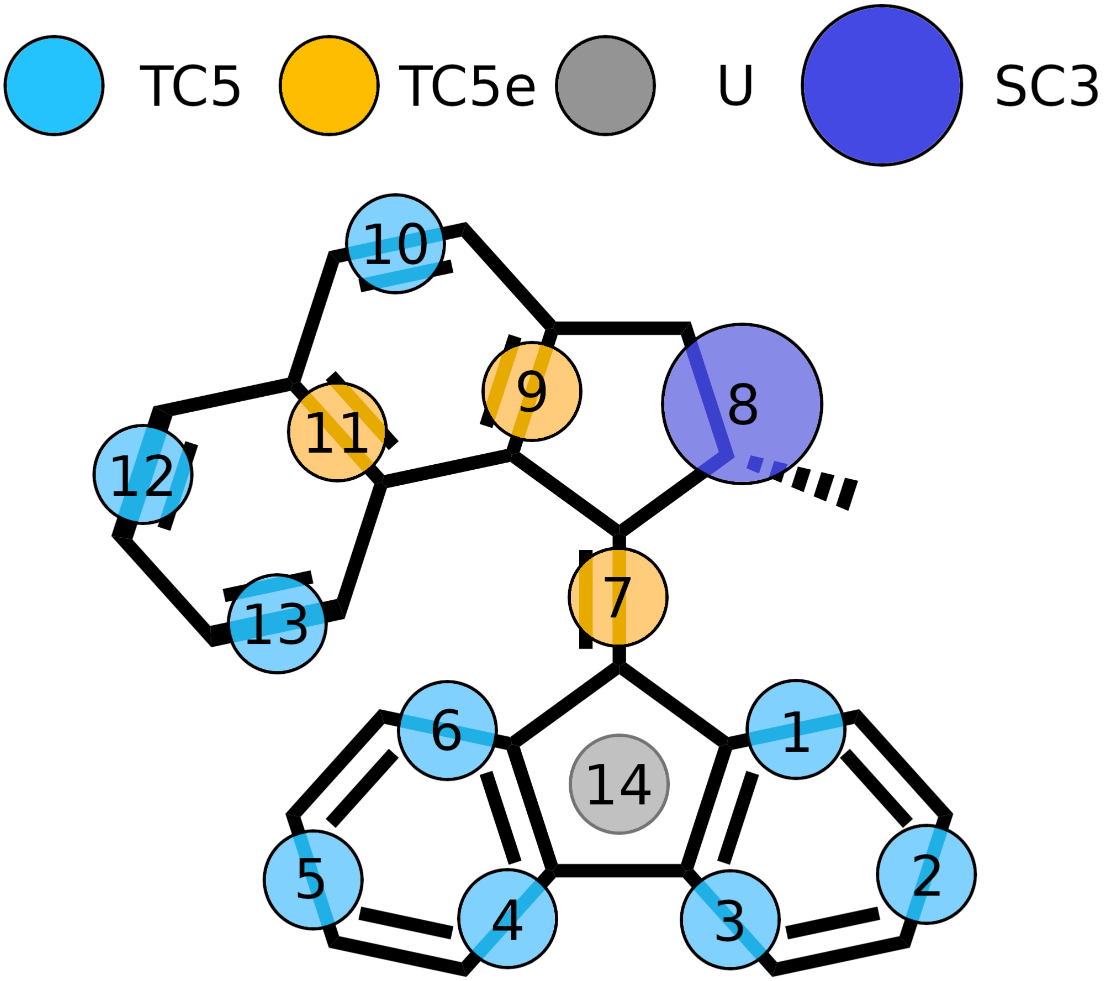

Figure S1: Mapping scheme of **MM1**.

Table S1: Bead types, charges and masses of tetrabutylammonium cation.

Number	Bead type	Charge	Mass
1	TC5	0	26
2	TC5	0	26
3	TC5	0	26
4	TC5	0	26
5	TC5	0	26
6	TC5	0	26
7	TC5e	0	24
8	SC3	0	42
9	TC5e	0	30
10	TC5	0	32
11	TC5e	0	0
12	TC5	0	32
13	TC5	0	32
14	U	0	0

Table S2: Virtual-site definitions of **MM1**.

virtual_sitesn		
**Site**	**Function**	**Constructing beads**
11	1	9 10 12 13
14	1	1 3 4 6

Table S3: Bonded parameters of **MM1**.

Parameter	i	j	k	l	Funct.	Ref.	Force c.	Multiplicity
Bond 1	1	7	-	-	1	0.245	3500	-
Bond 2	6	7	-	-	1	0.250	3500	-
Constraint 1	1	2	-	-	1	0.260	-	-
Constraint 2	1	3	-	-	1	0.310	-	-

Constraint 3	1	6	-	-	1	0.445	-	-
Constraint 4	2	3	-	-	1	0.260	-	-
Constraint 5	3	4	-	-	1	0.245	-	-
Constraint 6	4	5	-	-	1	0.260	-	-
Constraint 7	4	6	-	-	1	0.310	-	-
Constraint 8	5	6	-	-	1	0.260	-	-
Constraint 9	7	8	-	-	1	0.275	-	-
Constraint 10	7	9	-	-	1	0.245	-	-
Constraint 11	8	9	-	-	1	0.285	-	-
Constraint 12	9	10	-	-	1	0.255	-	-
Constraint 13	9	13	-	-	1	0.400	-	-
Constraint 14	10	12	-	-	1	0.415	-	-
Constraint 15	10	13	-	-	1	0.500	-	-
Constraint 16	12	13	-	-	1	0.295	-	-
Angle 1	14	7	9	-	1	155	150	-
Angle 2	5	14	2	-	1	140	250	-
Angle 3	1	3	4	-	1	110	150	-
Angle 4	6	4	3	-	1	110	150	-
Angle 5	7	9	10	-	1	165	150	-
Angle 6	5	4	3	-	1	165	250	-
Angle 7	2	3	4	-	1	165	250	-
Angle 8	1	7	8	-	1	130	150	-
Dihedral 1	1	2	6	5	2	180	200	-
Dihedral 2	4	3	6	1	2	-180	200	-
Dihedral 3	10	9	12	13	2	175	200	-
Dihedral 4	4	3	7	1	2	180	200	-
Dihedral 5	3	6	8	9	2	142	200	-

Dihedral 6	8	9	10	13	1	0	50	1
Dihedral 7	14	9	11	13	1	155	50	1

NOTES: All beads in the **stator** (beads 1-7 and 14) are excluded from one another, but not from the **rotor.** All beads in the **rotor** (beads 7-13) are excluded from one another, but not from the **stator**. The linker (bead 7) is excluded from all beads.

1.2. Molecular motor 2 (MM2)

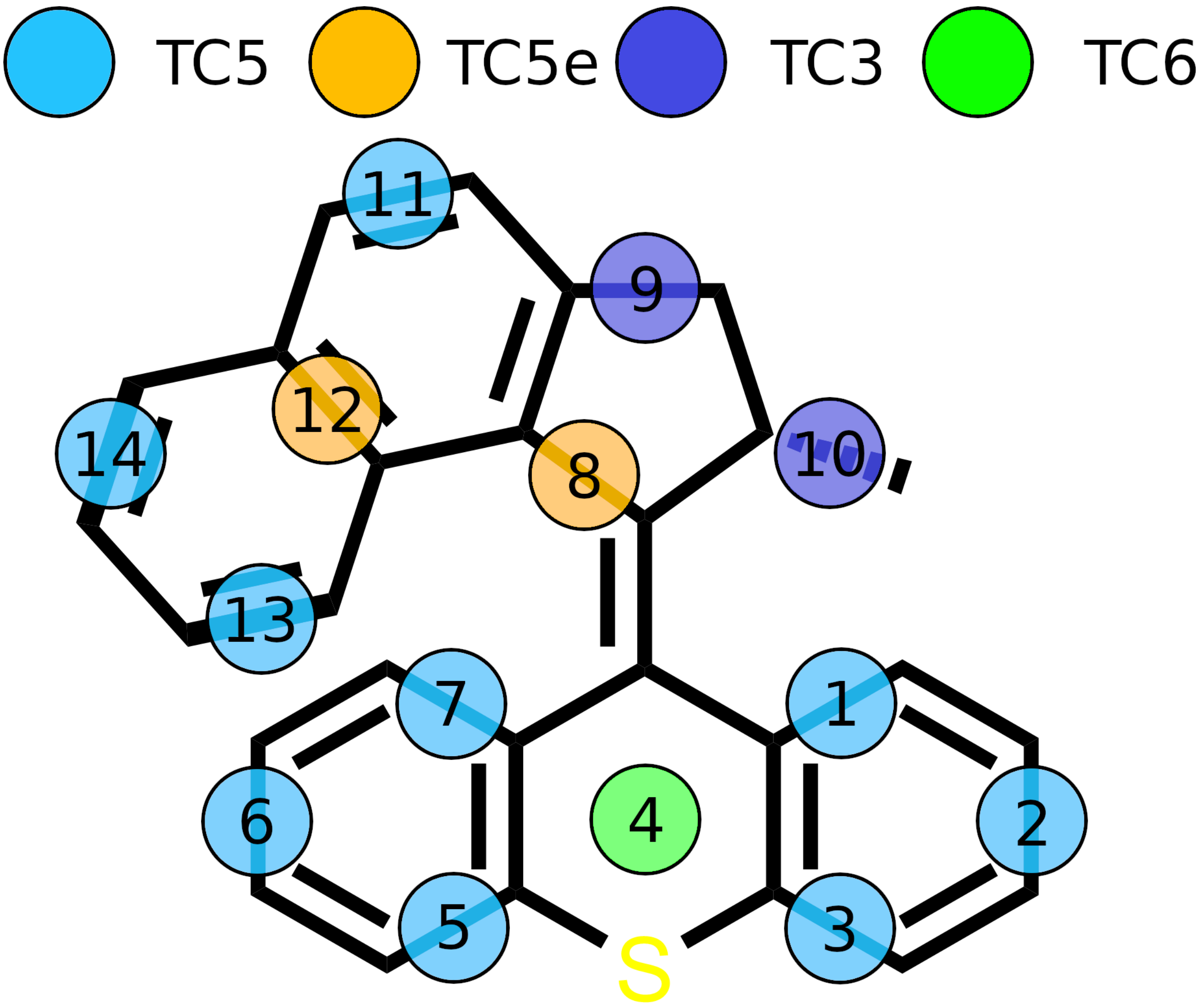

Figure S2: Mapping scheme of **MM2**.

Table S4: Bead types, charges and masses of **MM2**.

Number	Bead type	Charge	Mass
1	TC5	0	37
2	TC5e	0	0
3	TC5	0	59
4	TC6	0	0
5	TC5	0	37
6	TC5e	0	0
7	TC5	0	59
8	TC5e	0	24
9	TC3	0	26
10	TC3	0	29
11	TC5	0	38
12	TC5e	0	0
13	TC5	0	38
14	TC5	0	26

Table S5: Virtual-site definitions of **MM2**.

<table>
<tr><th colspan="5">virtual_sites2</th></tr>
<tr><th>Site</th><th>Constructing beads</th><th>Function</th><th colspan="2">a</th></tr>
<tr><td>4</td><td>3 7</td><td>1</td><td colspan="2">0.500</td></tr>
<tr><td>12</td><td>11 13</td><td>1</td><td colspan="2">0.500</td></tr>
<tr><th colspan="5">virtual_sites3</th></tr>
<tr><th>Site</th><th>Constructing beads</th><th>Function</th><th>a</th><th>b</th></tr>
<tr><td>2</td><td>1 3 4</td><td>1</td><td>1.010</td><td>1.010</td></tr>
<tr><td>6</td><td>4 5 7</td><td>1</td><td>1.010</td><td>1.010</td></tr>
</table>

Table S6: Bonded parameters of **MM2**.

Parameter	i	j	k	l	Funct.	Ref.	Force c.	Multiplicity
Bond 1	1	8	-	-	1	0.315	3500	-
Bond 2	7	8	-	-	1	0.325	3500	-
Constraint 1	1	3	-	-	1	0.305	-	-
Constraint 2	1	7	-	-	1	0.390	-	-
Constraint 3	3	5	-	-	1	0.430	-	-
Constraint 4	3	7	-	-	1	0.510	-	-
Constraint 5	5	7	-	-	1	0.305	-	-
Constraint 6	8	9	-	-	1	0.235	-	-
Constraint 7	8	11	-	-	1	0.365	-	-
Constraint 8	8	13	-	-	1	0.360	-	-
Constraint 9	9	10	-	-	1	0.260	-	-
Constraint 10	9	11	-	-	1	0.320	-	-
Constraint 11	11	13	-	-	1	0.510	-	-
Constraint 12	11	14	-	-	1	0.415	-	-
Constraint 13	13	14	-	-	1	0.295	-	-
Angle 1	8	9	10	-	1	65	50.0	-
Angle 2	5	7	8	-	1	110	100.0	-
**Quartic Angles**	**i**	**j**	**k**	**l**	**Funct.**	**Ref.**	**C_0, C_1, C_2, C_3, C_4**	
Angle 3	1	8	9	-	6	140	0, 0, -101, 0, 513	
Angle 4	7	8	11	-	6	145	0, 0, -131, 0, 538	
Angle 5	10	9	11	-	6	135	0, 0, -146, 0, 1065	
Dihedral 1	7	5	1	3	2	180	200	-
Dihedral 2	13	14	8	9	2	180	200	-
Dihedral 3	11	8	14	13	2	180	200	-
Dihedral 4	5	1	8	13	1	115	8	1

Dihedral 5	5	7	8	11	9	-75	18	1
Dihedral 6	5	7	8	11	9	17	12	2

NOTES: All beads in the **stator** (beads 1-7) are excluded from one another, but not from the **rotor.** All beads in the **rotor** (beads 8-14) are excluded from one another, but not from the **stator**.
Quartic Angles are used for bimodal angle distributions, and are defined by matching the distance between the two maxima and barrier heights.

1.3. Addend for molecular motor 1 (R1)

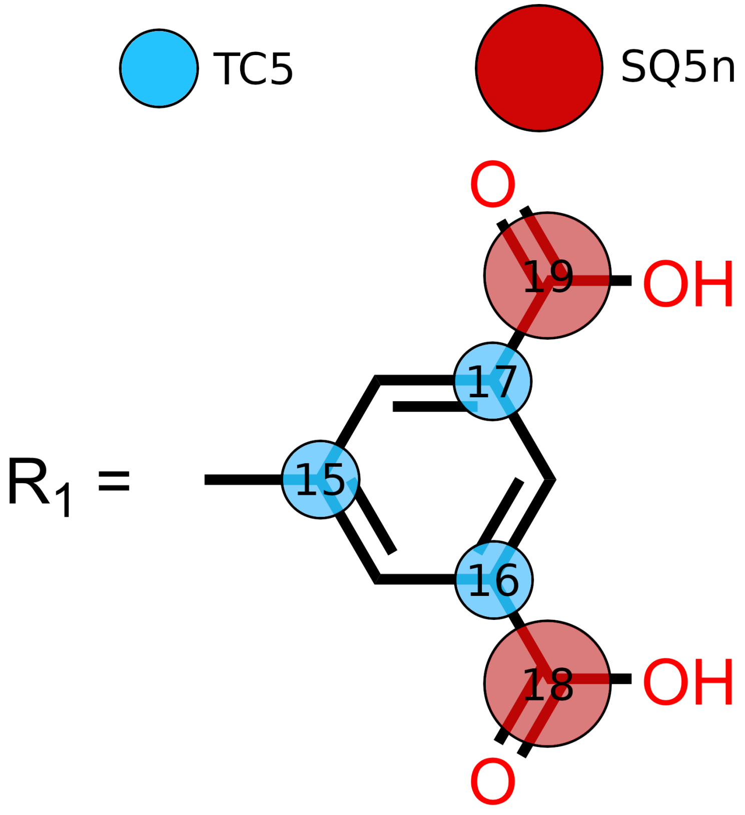

Figure S3: Mapping scheme of **R1**. Enumeration is carried on from **MM1** to help define the bonded parameters connecting **MM1** to **R1**.

Table S7: Bead types, charges and masses of **R1**.

Number	Bead type	Charge	Mass
15	TC5	0	25

16	TC5	0	25
17	TC5	0	25
18	SQ5n	-1	44
19	SQ5n	-1	44

Table S8: Bonded parameters of **R1**.

Parameter	i	j	k	l	Funct.	Ref.	Force c.	Multiplicity
Bond 1	15	16	-	-	1	0.200	3500	-
Bond 2	15	17	-	-	1	0.200	3500	-
Bond 3	16	17	-	-	1	0.200	3500	-
Bond 4	16	18	-	-	1	0.230	3500	-
Bond 5	17	19	-	-	1	0.230	3500	-
Angle 1	16	17	19	-	1	150	100	-
Angle 2	17	16	18	-	1	150	100	-
Angle 3	18	16	15	-	1	155	100	-
Angle 4	19	17	15	-	1	150	100	-

Table S9: Connection with **MM1** and **R1**. Numeration is from Figures **S1** and **S3**.

Parameter	i	j	k	l	Funct.	Ref.	Force c.	Multiplicity
Bond 1	2	15	-	-	1	0.240	3500	-
Angle 1	1	2	15	-	1	150	100	-
Angle 2	3	2	15	-	1	85	100	-

NOTES: Although both carboxylic acid groups of **R1** are drawn in their protonated state, the model is expected to reproduce the deprotonated state, which is used to electrostatically anchor **MM1** onto a positively charged surface.

1.4. Addend for molecular motor 2 (R2)

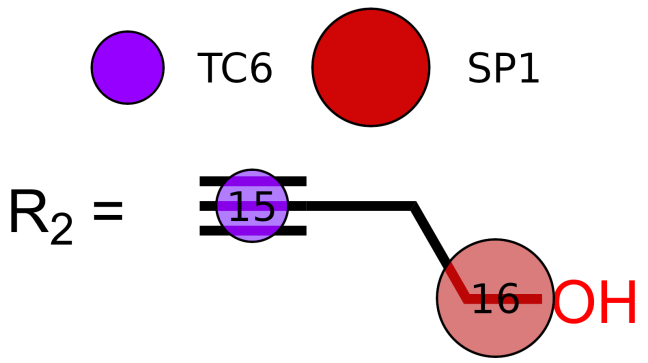

Figure S4: Mapping scheme of **R2**. Enumeration is carried on from **MM2** to help define the bonded parameters connecting **MM2** to **R2**.

Table S10: Bead types, charges and masses of **R2**.

Number	Bead type	Charge	Mass
15	TC6	0	24
16	SP1	0	58

Table S11: Bonded parameters of **R2**.

Parameter	i	j	k	l	Funct.	Ref.	Force c.	Multiplicity
Bond 1	15	16	-	-	1	0.275	3500	-

Table S12: Connection with **MM2** and **R2**. Numeration is from Figures **S2** and **S4**.

Parameter	i	j	k	l	Funct.	Ref.	Force c.	Multiplicity
Bond 1	2	15	-	-	1	0.260	3500	-
Angle 1	3	2	15	-	1	155	200	-
Angle 2	2	15	16	-	1	145	25	-
Dihedral 1	7	1	2	15	1	30	12	1

NOTES:

1.5. Molecular switch, E-stereoisomer (MS-E)

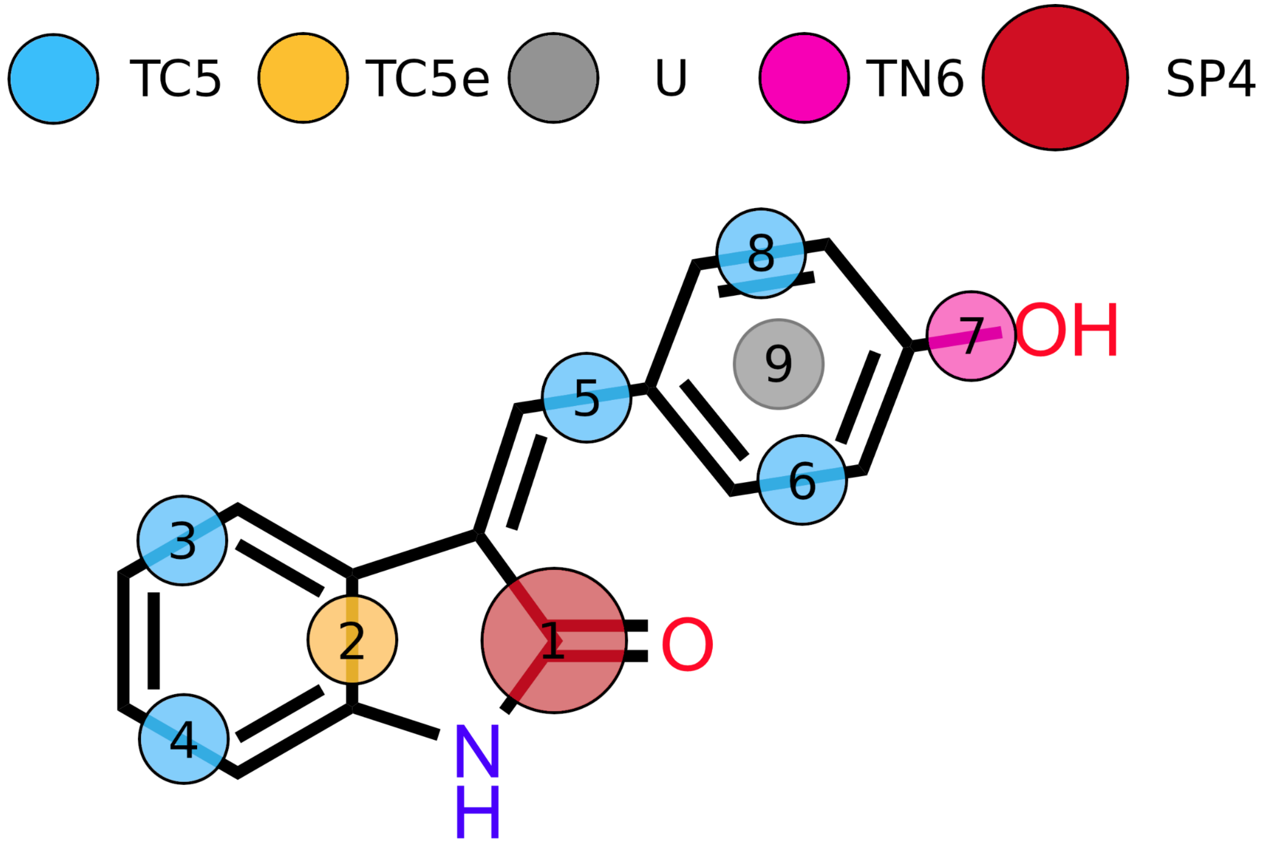

Figure S5: Mapping scheme of **MS-E**.

Table S13: Bead types, charges and masses of **MS-E**.

Number	Bead type	Charge	Mass
1	SP4	0	64
2	TC5e	0	0
3	TC5	0	35
4	TC5	0	35
5	TC5	0	24
6	TC5	0	24
7	TN6	0	30
8	TC5	0	24
9	U	0	0

Table S14: Virtual-site definitions of **MS-E**.

virtual_sites2				
**Site**	**Constructing beads**	**Function**	**a**	
9	6 8	1	0.500	
**virtual_sites3**				
**Site**	**Constructing beads**	**Function**	**a**	**d**
2	1 3 4	2	0.650	0.200

Table S15: Bonded parameters of **MS-E**.

Parameter	i	j	k	l	Funct.	Ref.	Force c.	Multiplicity
Constraint 1	1	4	-	-	1	0.415	-	-
Constraint 2	1	5	-	-	1	0.330	-	-
Constraint 3	3	1	-	-	1	0.295	-	-
Constraint 4	3	4	-	-	1	0.455	-	-
Constraint 5	3	5	-	-	1	0.450	-	-
Constraint 6	5	6	-	-	1	0.325	-	-
Constraint 7	5	8	-	-	1	0.325	-	-
Constraint 8	6	7	-	-	1	0.315	-	-
Constraint 9	6	8	-	-	1	0.335	-	-
Constraint 10	7	8	-	-	1	0.315	-	-
Angle 1	3	5	9	-	1	85	250	-
Dihedral 1	1	5	4	3	2	180	150	-
Dihedral 2	6	7	5	8	2	180	150	-
Dihedral 3	3	5	6	7	1	0	4	4
Dihedral 4	4	3	5	9	9	180	4	2
Dihedral 5	4	3	5	9	9	0	16	1

NOTES: Both virtual sites are excluded from their surrounding beads.

1.6. Molecular switch, Z-stereoisomer (MS-Z)

MS-Z is identical to **MS-E** in terms of bead assignments and virtual site constructions. The differences between the stereoisomers are in the bonded terms, which are given in Table S16 below.

Table S16: Bonded parameters of **MS-Z**.

Parameter	i	j	k	l	Funct.	Ref.	Force c.	Multiplicity
Constraint 1	1	4	-	-	1	0.415	-	-
Constraint 2	1	5	-	-	1	0.350	-	-
Constraint 3	3	1	-	-	1	0.450	-	-
Constraint 4	3	4	-	-	1	0.295	-	-
Constraint 5	3	5	-	-	1	0.425	-	-
Constraint 6	5	6	-	-	1	0.325	-	-
Constraint 7	5	8	-	-	1	0.325	-	-
Constraint 8	6	7	-	-	1	0.315	-	-
Constraint 9	6	8	-	-	1	0.335	-	-
Constraint 10	7	8	-	-	1	0.315	-	-
Angle 1	1	5	9	-	1	115	250	-
Dihedral 1	1	5	4	3	2	180	150	-
Dihedral 2	6	7	5	8	2	180	150	-
Dihedral 3	1	5	6	7	1	180	15	2
Dihedral 4	4	1	5	6	9	0	50	1

NOTES: Both virtual sites are excluded from their surrounding beads.

1.7. Calcein

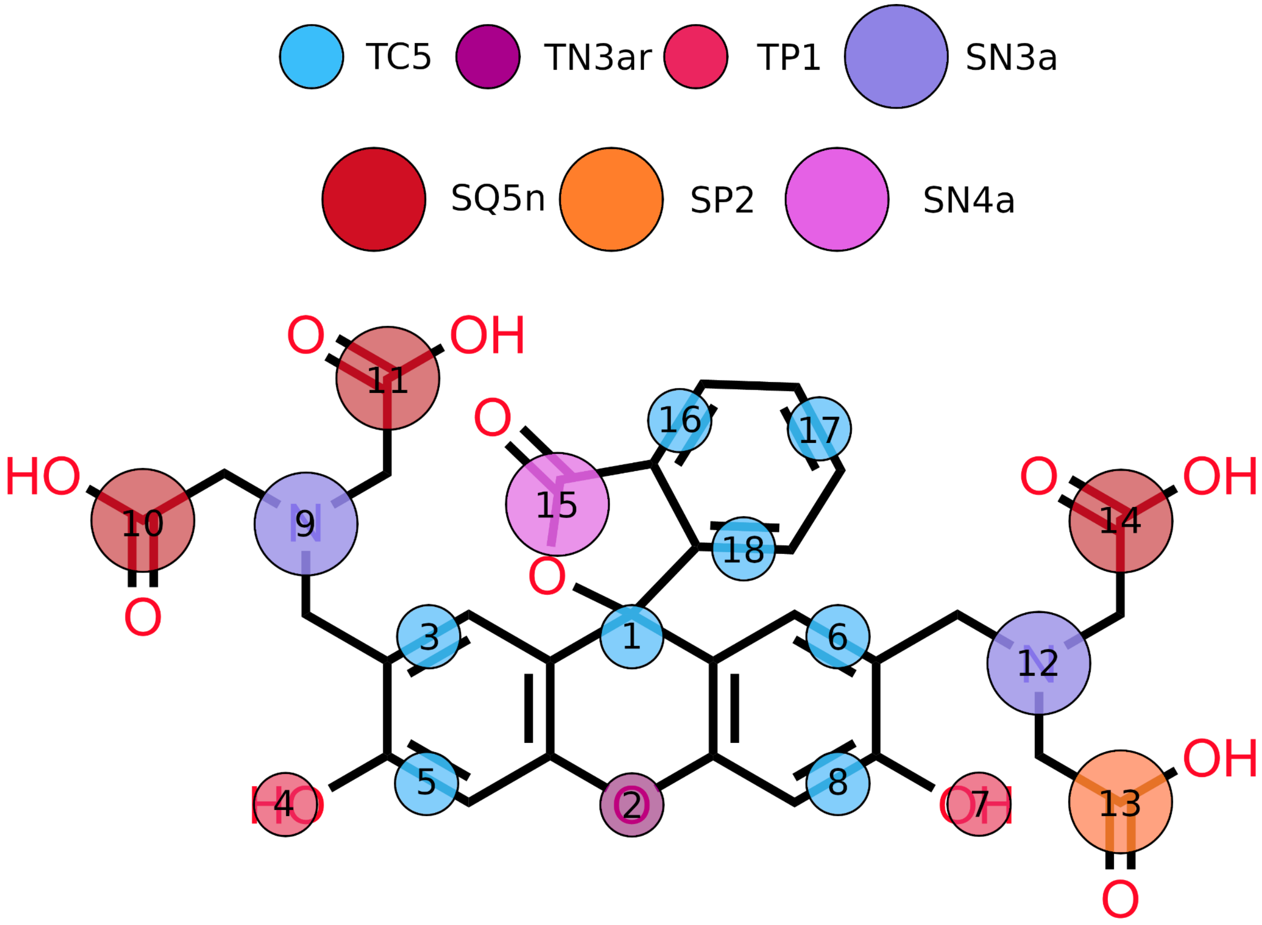

Figure S6: Mapping scheme of calcein.

Table S17: Bead types, charges and masses of calcein.

Number	Bead type	Charge	Mass
1	TC5	0	24
2	TN3ar	0	28
3	TC5	0	25
4	TP1	0	29
5	TC5	0	25
6	TC5	0	25
7	TP1	0	29
8	TC5	0	25

9	SN3a	0	56
10	SQ5n	-1	44
11	SQ5n	-1	44
12	SN3a	0	56
13	SP2	0	45
14	SQ5n	-1	44
15	SN4a	0	44
16	TC5	0	25
17	TC5	0	26
18	TC5	0	25

Table S18: Bonded parameters of calcein.

Parameter	i	j	k	l	Funct.	Ref.	Force c.	Multiplicity
Bond 1	3	9	-	-	1	0.310	3000	-
Bond 2	6	12	-	-	1	0.310	3000	-
Bond 3	9	10	-	-	1	0.290	3000	-
Bond 4	9	11	-	-	1	0.270	3000	-
Bond 5	12	13	-	-	1	0.290	3000	-
Bond 6	12	14	-	-	1	0.270	3000	-
Constraint 1	1	3	-	-	1	0.260	-	-
Constraint 2	1	6	-	-	1	0.260	-	-
Constraint 3	2	5	-	-	1	0.240	-	-
Constraint 4	2	8	-	-	1	0.240	-	-
Constraint 5	3	5	-	-	1	0.305	-	-
Constraint 6	4	5	-	-	1	0.235	-	-
Constraint 7	6	8	-	-	1	0.305	-	-
Constraint 8	7	8	-	-	1	0.235	-	-

Constraint 9	1	15	-	-	1	0.280	-	-
Constraint 10	1	18	-	-	1	0.240	-	-
Constraint 11	15	16	-	-	1	0.265	-	-
Constraint 12	16	17	-	-	1	0.260	-	-
Constraint 13	16	18	-	-	1	0.310	-	-
Constraint 14	17	18	-	-	1	0.260	-	-
Angle 1	10	9	11	-	1	116	200	-
Angle 2	13	12	14	-	1	116	200	-
Angle 3	11	9	3	-	1	145	25	-
Angle 4	13	12	6	-	1	145	25	-
Angle 5	9	3	1	-	1	160	200	-
Angle 6	12	6	1	-	1	160	200	-
Angle 7	9	3	5	-	1	110	100	-
Angle 8	12	6	8	-	1	110	100	-
Angle 9	3	5	4	-	1	70	200	-
Angle 10	6	8	7	-	1	70	200	-
Angle 11	3	5	2	-	1	80	200	-
Angle 12	6	8	2	-	1	80	200	-
Angle 13	3	1	6	-	1	165	200	-
Angle 14	5	2	8	-	1	155	200	-
Angle 15	15	1	18	-	1	95	100	-
Angle 16	15	16	17	-	1	135	100	-
Angle 17	16	17	18	-	1	70	100	-
Angle 18	17	18	1	-	1	145	100	-
Dihedral 1	3	5	1	2	2	180	200	-
Dihedral 2	8	6	2	1	2	180	200	-
Dihedral 3	4	5	3	2	2	180	200	-

Dihedral 4	7	8	6	2	2	180	200	-
Dihedral 5	15	18	16	17	2	180	200	-
Dihedral 6	6	1	18	15	2	85	200	-
Dihedral 7	3	1	15	17	2	85	200	-

NOTES: Beads 1-7 are excluded from one another, as are beads 15-18. Structure of calcein is presented in the fully protonated state, but the model is built to reproduce calcein at pH ~ 7.4, in which it carries a charge of -3. This state is depicted by the three SQ5n beads and the one SP2 bead.

2. Solvent Accessible Surface Areas (SASAs)

SASAs were computed for all AA and CG models used in this study. **FMM1** and **FMM2** refer to the functionalized molecular motors (motors with addends). The SASA was estimated with the *gmx sasa* tool which comes standard with GROMACS installations. We used a probe radius of 0.191 nm and 4800 dots per sphere for both resolutions. Analysis on the resulting distribution was performed with *gmx analyze*, using a bin width of 0.01. Atomic radii used to evaluate the AA models were taken from Rowland *et al.*[1] Results of the analysis are given in Table S19.

Table S19: Measured SASA values for each CG model created for this study, and its AA counterpart. Positive difference indicates the Martini model to be larger than the atomistic model, and vice versa.

Compound	Martini 3 (nm²)	All atom (nm²)	Difference (%)
**MM1**	6.66 ± 0.12	6.52 ± 0.03	2.14
**FMM1**	10.72 ± 0.45	11.39 ± 0.10	-5.88
**MM2**	6.71 ± 0.09	6.94 ± 0.03	-3.31
**FMM2**	9.14 ± 0.34	9.70 ± 0.18	-5.77
**MS-E**	5.42 ± 0.07	5.36 ± 0.02	1.12
**MS-Z**	5.53 ± 0.08	5.39 ± 0.02	2.60
**Calcein**	9.31 ± 0.31	9.45 ± 0.18	-1.48

[1] **Rowland, R. S.; Taylor, R.** Intermolecular Nonbonded Contact Distances in Organic Crystal Structures: Comparison with Distances Expected from van der Waals Radii. *The Journal of Physical Chemistry* 1996, 100, 7384–7391

3. Comparison of the key bonded terms

The most important bonded interaction for both the MMs and MSs are the dihedral terms, which, in the case of MMs dictate the orientation of the rotor in relation to the stator, and in the case of MSs controls the stereoisomerism. Dihedral profiles for MM1, MM2, MS-E and MS-Z are illustrated in Figure S7.

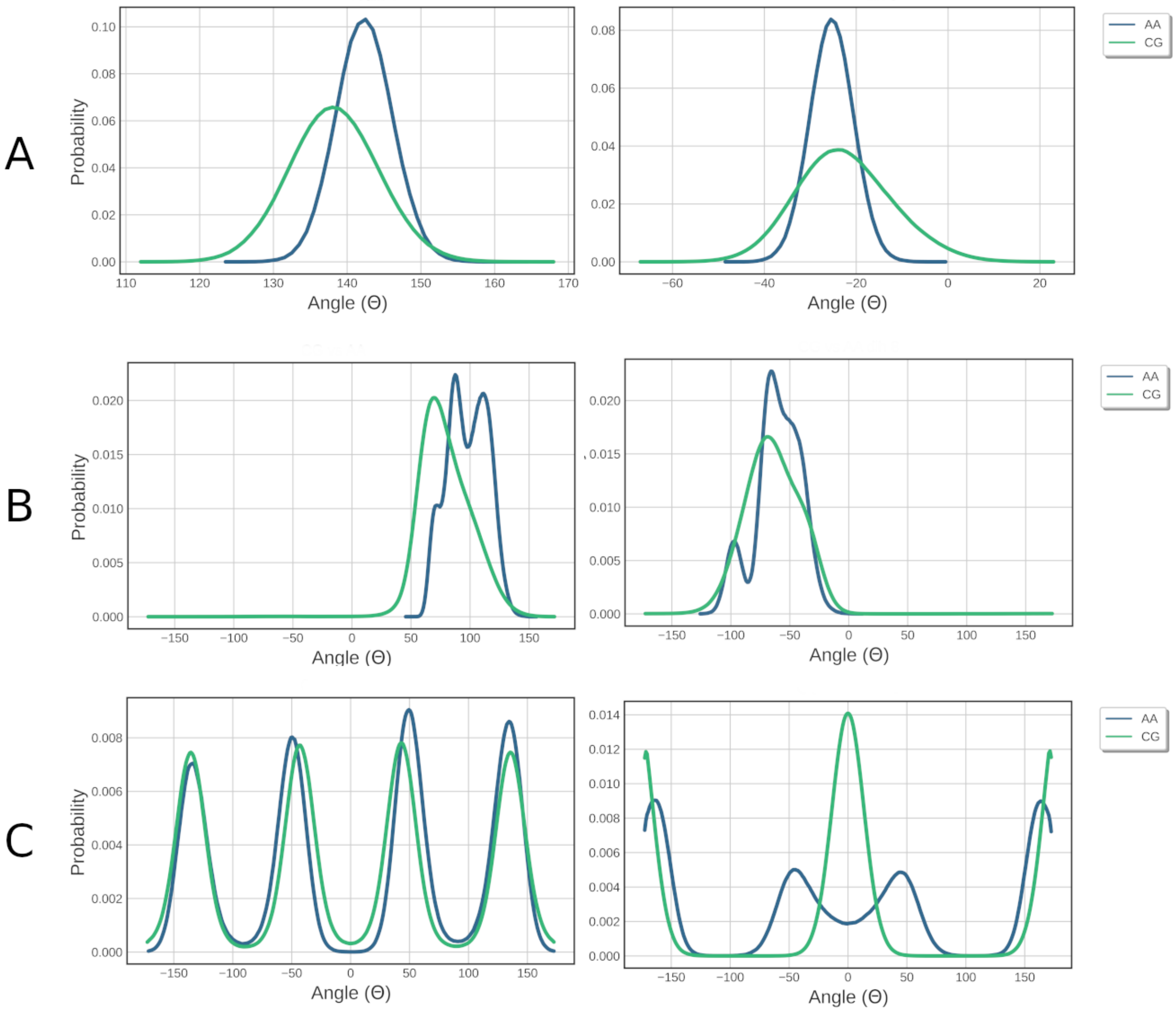

Figure S7: Dihedral profiles. A) MM1, two dihedrals are used to define the orientation of rotor in relation to the stator. B) MM2, same as MM1. C) Left: MS-E, tetramodal dihedral between beads 1-4 and 5-9. Right: MS-Z, same dihedral as with MS-E.
